# Risk Factors for Obesity at Age 3 in Alaskan Children, Including the Role of Beverage Consumption: Results from Alaska PRAMS 2005-2006 and Its Three-Year Follow-Up Survey, CUBS, 2008-2009

**DOI:** 10.1371/journal.pone.0118711

**Published:** 2015-03-20

**Authors:** Janet M. Wojcicki, Margaret B. Young, Katherine A. Perham-Hester, Peter de Schweinitz, Bradford D. Gessner

**Affiliations:** 1 Department of Pediatrics (GI and Nutrition), University of California San Francisco, San Francisco, California, United States of America; 2 Maternal and Child Health Epidemiology Unit, Alaska Division of Public Health, Section of Women's, Children's and Family Health, Anchorage, Alaska, United States of America; 3 Tanana Chiefs Conference, Fairbanks, Alaska, United States of America; 4 Agence de Medicin Preventive, Paris, France; McMaster University, CANADA

## Abstract

**Background:**

Prenatal and early life risk factors are associated with childhood obesity. Alaska Native children have one of the highest prevalences of childhood obesity of all US racial/ethnic groups.

**Methods:**

Using the Pregnancy Risk Assessment Monitoring System (PRAMS) and the follow-up survey at 3 years of age (CUBS), we evaluated health, behavioral, lifestyle and nutritional variables in relation to obesity (95th percentile for body mass index (BMI)) at 3 years of age. Multivariate logistic regression modeling was conducted using Stata 12.0 to evaluate independent risk factors for obesity in non-Native and Alaska Native children.

**Results:**

We found an obesity prevalence of 24.9% in all Alaskan and 42.2% in Alaska Native 3 year olds. Among Alaska Native children, obesity prevalence was highest in the Northern/Southwest part of the state (51.6%, 95%CI (42.6-60.5)). Independent predictive factors for obesity at age 3 years in Alaska non-Native children were low income (<$10,000 in the year before the child was born (OR 3.94, 95%CI 1.22--17.03) and maternal pre-pregnancy obesity (OR 2.01, 95%CI 1.01-4.01) and longer duration of breastfeeding was protective (OR 0.95, 95%CI 0.91-0.995). Among Alaska Native children, predictive factors were witnessing domestic violence/abuse as a 3 year-old (OR 2.28, 95%CI 1.17-7.60). Among obese Alaska Native children, there was an increased daily consumption of energy dense beverages in the Northern/Southwest region of the state, which may explain higher rates of obesity in this part of the state.

**Conclusions:**

The high prevalence of obesity in Alaska Native children may be explained by differences in lifestyle patterns and food consumption in certain parts of the state, specifically the Northern/Southwest region, which have higher consumption of energy dense beverages.

## Background

Childhood overweight and obesity affect 31.8% and 16.9%, respectively, of American youth ages 2–19 years [1] (Ogden et al., 2012). While overweight and obesity are problems in all communities in the United States, American Indian and Alaska Native (AI/AN) groups have the highest prevalence among US racial groups. A study with nationally representative preschool-age children found that 31.2% of AI/AN children were obese at age 4 years, with the prevalence among all US children being 18.4% [2]. In Alaska, approximately one third of children entering kindergarten and first grade in the Anchorage School District (ASD) are overweight or obese [3]; 36% of pre-K to 12^th^ grade students in the Kenai Peninsula Borough School District are overweight or obese [4]; and close to 20% of students in grades 5 and 7 in two Anchorage-area school districts are obese [5]. The prevalence of overweight and obesity among low income children ages 2–4 years participating in the Special Supplemental Nutrition Program for Women, Infants and Children (WIC) in Alaska is similarly high at 40% [3].

As the problem of obesity in children starts early and there are few interventions that successfully treat obesity, some have focused prevention efforts on the prenatal period and first year of life [6]. Previous studies have identified in utero and early life risk factors that increase future risk for childhood obesity. These risk factors include maternal tobacco cigarette use during pregnancy [7], excessive maternal prenatal weight gain [8,9], shorter duration of breastfeeding, and, possibly, maternal postpartum depressive symptoms [10]. For preschool children, increased sugar-sweetened beverage consumption has been associated with obesity [11], and previous data from Alaska has found a high overall consumption of sugar-sweetened beverages (SSBs), with 31% of 3-year-olds drinking SSBs daily [12].

Using data from the Alaska Pregnancy Risk Assessment Monitoring System (PRAMS) and the follow-up of this cohort titled the Alaska Childhood Understanding Behaviors Survey (CUBS), we sought to evaluate the potential role of exposures just prior to pregnancy, during pregnancy, in early infancy and before age 3 years with risk for obesity at age 3 years. In particular, we were interested to evaluate the role of previous known risk factors for obesity in this population including maternal pre-pregnancy obesity, SSB consumption including 100% fruit juice consumption and the protective role of breastfeeding. Given the high prevalence of obesity among Alaska Native children, we also sought to delineate risk factors for Alaska Native children and compare these risk factors with those for non-Native Alaska children to better understand what places Alaska Native children at higher risk.

## Methods

The authors used a retrospective observational cohort study design, linking weighted data for Alaska PRAMS 2005–2006 birth year respondents (Phase 5 survey) to CUBS respondents in 2008 (Phase 2) and 2009 (Phase 3). PRAMS is population-based survey, which provides weighted estimates for sample design, nonresponse and non-coverage to create statewide estimates to accurately reflect Alaska’s population demographics [13].

### Data Sources

1. Pregnancy Risk Assessment Monitoring System: PRAMS is a retrospective and cross-sectional population based survey, which collects maternal self-reported information on attitudes and experiences before, during, and after the delivery of a live-born infant with information collected at one time-point during the postpartum period (approximately 2 to 6 months after delivery). Alaska PRAMS samples about one of every six live births through a stratified random sample of birth certificates, with stratification conducted according to maternal Alaska Native race and infant birth weight. All sampled respondents are linked to birth certificate information. The PRAMS unweighted response rates (or raw number not adjusting for Alaska’s population demographics) for 2005 and 2006 were 77% and 75%, respectively. The full methodology of PRAMS is described elsewhere [14]. We had 2,802 mothers who participated in PRAMS in 2005 and 2006.
2. Alaska Childhood Understanding Behaviors Survey (CUBS): CUBS collects data about the health status and care of Alaskan children at 3 years of age. Mothers who completed a PRAMS survey and are still living in Alaska receive up to two copies of the CUBS survey in the mail soon after their child’s third birthday. PRAMS phone respondents whose child was born in the last six months of 2006 also had the option of completing the CUBS survey by phone. The CUBS survey includes questions on the child’s current height and weight, nutrition and eating habits, health care utilization and access, and child development and behaviors as previously described [15]. CUBS unweighted response rates among women eligible in 2008 and 2009 were 49% and 50% (or 1,136 of the 2,802).

### Data analysis

Our outcome was obesity defined as BMI ≥95^th^ percentile (age and sex specific) at age 3 years and was calculated from the child’s height and weight as self-reported by the mother on CUBS. All non-obese children, including those who were underweight (5.2% of non-Native children and 3.5% of Alaska Native children), were compared to obese children.

We analyzed obesity by maternal Alaska Native vs. non-Native race. The terms “Alaska Native children” and “non-Native children” are based on maternal race only, as reported on the birth certificate. Risk factors for childhood obesity were assessed using t-tests for continuous variables and chi-square tests for categorical variables. Pre-pregnancy, prenatal, postnatal (from 2–8 months postpartum), and childhood predictors at age 3 years were included for evaluation of risk for obesity. Variables that were significant at p<0.05 in bivariate analysis were included in the multivariate model. When variables were highly correlated with one another with r>0.5 (such as prenatal and postnatal maternal cigarette smoking, income status from the time of the birth of the child and at 3 years of age, and location of residence and urban/rural) we included in multivariate analysis either the variable more strongly associated with obesity in bivariate analysis or the variable most important to elucidate obesity trends in the state of Alaska (region of residence).

Variables that were known from previous studies to be associated with increased risk for obesity in early childhood were also included in the multivariate model process including maternal pre-pregnancy weight category (obese versus non-obese), breastfeeding duration (continuous), maternal age (continuous) and consumption of sugar-sweetened beverages including 100% fruit juice. Beverage frequency variables were analyzed both ordinally (how the questions were originally asked on the CUBS questionnaire), as well as dichotomously, with the cut point used corresponding to the approximate mean or median of the variable for the population. The cut point for beverages was either ≥1 cup/day or >1 cup/day. Food consumption questions were analyzed dichotomously with the cut point similarly corresponding to the approximate mean, with cut-points reflective of the number of times per day the item was consumed. All food and beverage questions were based on questions that asked about consumption yesterday with the exception of the milk consumption question, which asked about usual consumption.

Because of the high percentage of missing data for paternal education (10.5%), we did not include paternal education in the multivariate models even though they were significant in bivariate <0.05 for Alaska Native children. Due to a high percentage of respondents who were missing data on child BMI (16.5%), we compared the percentage of selective socio-demographic factors (maternal age, paternal education, maternal education and location of residence) in those who were missing for BMI compared with non-missing participants (for BMI) (as 16.5% was also missing for BMI).

Subsequently, an analysis was conducted to compare daily consumption of milk, 100% fruit juice, soda and SSB, as well as usual type of milk child drank, based on regional residence (Anchorage, Interior, Northern/Southwest and Gulf Coast/Southeast). This analysis was conducted so as to better understand the protective association found between fruit juice consumption and obesity in multivariate analysis for Alaska Native children only and not non-Native children. Residence was obtained from the mother’s address at the time she completed the CUBS survey, and regional groups were a modified version of those defined by the Alaska Department of Labor (http://labor.alaska.gov/research/index.htm)—Northern and Southwest regions, and Gulf Coast and Southeast regions, respectively, were combined due to small numbers and because these regions are geographically contiguous. We also evaluated associations between 100% fruit juice consumption and consumption of other beverages and food using chi-squared tests to better understand the protective relationship between 100% fruit juice consumption and obesity. Additionally, we examined differences in bivariate associations for non-Native and Alaska Native children using an interaction term between the predictor of interest and being either Native or Non-Native and child obesity at age 3 in logistic models. All data analysis used Stata 13.0 using “svy” statements to allow for the sampling weights provided by PRAMS. Sampling weights are necessary in survey analysis to get the survey weighting, clustering and stratification accounted for so as to get standard errors of estimates correct. Data are expressed as means ±standard errors (SE). Specific details about the PRAMS and CUBS variables used in the analysis are included as a supplement to this article (S1 Appendix: Additional Methodological Details on PRAMS and CUBS Variables).

This was primarily a hypothesis-generating study and we did not do any multiple comparisons to reduce the chance of Type II errors [16].

## Results

The mean age of the children when the PRAMS survey was conducted was 118.1 (standard error [SE], ±28.6) days (range 68–243) or approximately 4 months of age with a range from 2 to 8 months. The mean age when the CUBS survey was conducted was 36.8±1.0 months (range 35–42). The total number of children followed to 3 years was 1,136 as part of CUBS with 947 (83.5%) having a non-missing weight and height measurement for the child thus allowing calculation of BMI and obesity and 833 (73.3%) having a non-missing BMI and race/ethnicity (Table 1). Basic frequencies of selected variables for the cohort as a whole and stratified by Alaska Native and non-Native race are presented in Table 1. Obesity prevalence among all 3 year olds was 24.9%, 95%CI (21.5–28.6) and among Alaska Native children 42.2%, 95%CI (36.3–48.4) (Table 1), similar to what was previously reported for CUBS 2009 (Alaska Department of Health and Social Services, 2012).

**Table 1:** Weighted Frequencies of selected variables in PRAMS and CUBS Sample (n = 833).

	Total population % or mean	Alaska Native* (% or mean±SE)	Alaska Non-Native* (% or mean±SE)
		(N = 277)	(N = 556)
Maternal and paternal specific variables			
Socio-demographics			
Alaska Native	22.1		
Alaska Non-Native	77.9		
Maternal Age	27.8±0.25	26.1± 0.38	28.2±0.31
Place of Residence			
Anchorage (region)	51.3	23.9	58.4
Interior	16.6	13.9	17.5
Northern/Southwest	11.6	46.9	1.7
Gulf Coast/Southeast	20.5	15.3	22.4
Maternal Education			
<12 years	10.3	22.8	6.7
12 years	42.8	52.5	40.2
>12 years	45.4	22.7	51.9
Missing	1.5	2.1	1.1
Paternal Education			
<12 years	6.8	11.1	5.5
12 years	42.7	53.9	39.6
>12 years	40.0	15.9	46.8
Missing	10.6	19.1	8.1
Married at birth			
Yes	72.2	52.7	77.4
No	27.8	47.3	22.6
Maternal Pre-pregnancy body mass index (BMI) category (kg/m ^2^ )			
Underweight <18.5	3.1	1.7	3.5
Normal ≥18.5 & <25	51.4	41.0	54.4
Overweight ≥25 & <30	25.3	29.4	24.0
Obese ≥30	20.3	27.8	18.2
Child BMI Category			
Obese ≥95^th^ %ile	24.9	42.2	19.9
Overweight <95 & ≥85^th^%ile	16.0	15.9	16.1
Normal Weight<85^th^ & ≥5^th^%ile	54.3	38.4	58.9
Underweight <5^th^ %ile	4.8	3.5	5.2
BMI	18.1±0.2	17.4±0.4	18.3±0.2

*Note: As determined by maternal race.

Among Alaska Native 3 year olds, the Northern/Southwest had the highest obesity prevalence (51.6%, 95%CI (42.6–60.5)) (Table 2; S1 Fig. for visual image of different regions).

**Table 2:** Percent Obese at Age 3 (≥95^th^ percentile) by Maternal Socio-demographic Variables.

Variable	Non-Native Population	p-value	Alaska Native Population	p-value	p-value comparing
	Obese ≥ 95 ^th^ %ile (N = 96)		Obese ≥ 95 ^th^ %ile (N = 109)		groups
Place of Current Residence					
Anchorage (region)	16.8	0.24	32.4	0.04	0.04
Interior	25.5		38.2		
Northern/Southwest	12.9		51.6		
Gulf Coast/Southeast	24.9		32.3		
Urban/Rural Residence at Time of Birth					
Urban residence	16.1	0.40	32.7	0.02	
Rural residence	21.0		47.4		
Maternal Race					
White	18.0				
Non-White	33.1	0.04			
Maternal and Paternal Education and Age at Time of Child’s Birth					
Maternal Education					
<12 years	23.4	0.55	47.9	0.06	0.48
12 years	21.7		46.2		
>12 years	18.4		26.6		
Missing	4.14		47.5		
Paternal Education					
<12 years	28.6	0.014	55.2	0.02	0.74
12 years	22.7		43.8		
>12 years	13.5		21.4		
Missing	36.6		47.3		
Maternal Age					
≤19 years	13.3	0.43	60.7	0.16	0.73
20–24	24.4		41.1		
25–29	17.5		38.4		
30–34	24.5		34.0		
≥35	15.6		44.6		
Marital Status at time of child’s birth					
Married (yes)	16.0	<0.01	39.4	0.44	0.10
Married (no)	31.0		44.3		
Income, 12 mos before child born					
<$10,000	47.6	<0.01	38.8	0.45	<0.01
≥$10,000	17.7		44.1		
Income when child is 3 years of age (last 12 months)					
<$17,500	24.8	0.51	33.1	0.57	
≥$17,500	20.0		11.2		ND
Prenatal WIC participation					
Yes	21.5	0.57	47.1	0.02	0.19
No	19.0		31.5		

### Childhood Obesity Risk Factors in Alaska non-Native Children

#### Maternal and Family Socio-Demographic Variables

Among non-Native children, maternal and family socio-demographic variables that were associated with risk for childhood obesity at age 3 years included non-white maternal race (33.1% versus 18.0%; p = 0.04, Table 2) and less paternal education (<12 years versus >12 years; 28.6% versus 13.5% and 22.7% for 12 years; p = 0.014). Not being married compared to being married at the time of the child’s birth was associated with increased risk for obesity (31.0% versus 16.0%; p<0.01) as was total income in the 12 months before the child was born (p<0.01) but not when the child was 3 years of age (p = 0.51; Table 2). Income by number of dependents was lower at age 3 (in prior 12 months) in relation to child’s obesity ($11,547.53±720.32 for obesity versus $13,001.10±362.80 for non-obese, p = 0.07; results not shown) and in the 12 months prior to the child’s birth ($12,216.4±895.86 for obesity versus $14,735.82±488.81 for non-obese; p = 0.01; results not shown).

#### Prenatal and postnatal health

We found few differences in the prevalence of obesity among 3-year-olds based on the prenatal health of mothers. Factors most strongly correlated with obesity included smoking during the postpartum period (32.5% versus 18.0%; p = 0.02) (Table 3), >40 lbs maternal weight gain during pregnancy (24.4% versus 16.0%; p = 0.06) and maternal smoking 3 months before pregnancy (28.1% versus 17.6%; p = 0.06) (Table 3), although the last two factors were not statistically significant but trended towards significance.

**Table 3:** Percent Obese at Age 3 (≥95^th^ percentile) by Maternal Prenatal/ Postnatal Health Variables and Infant Feeding/Delivery Variables.

Variables	Non-Native Population Obese ≥ 95 ^th^ %ile (N = 96)	p-value	Alaska Native Population Obese ≥ 95 ^th^ %ile (N = 109)	p-value	p-value comparing groups
Gestational diabetes mellitus					
Yes	28.9	0.26	29.1	0.23	0.10
No	19.1		43.1		
Pre-existing diabetes mellitus					
Yes	24.5	0.76	40.2	0.89	0.06
No	19.6		42.3		
>40 lbs wt gain in pregnancy					
Yes	24.4	0.06	41.1	0.20	0.21
No	16.0		43.8		
Mom Pre-pregnancy Body Mass Index (BMI) category (kg/m ^2^ )					
Underweight	24.6	0.09	34.7	0.35	0.63
<18.5					
Normal	16.3		37.5		
≥18.5 & <25					
Overweight	19.4		40.6		
≥ 25 & <30					
Obese ≥30	31.1		51.0		
Mom smoked 3 months before pregnancy					
Yes	28.1	0.06	45.7	0.32	0.89
No	17.6		39.3		
Mom smoked during pregnancy					
Yes	24.7	0.47	50.0	0.19	0.38
No	19.1		40.0		
Mom smoked in post-partum					
Yes	32.5	0.02	48.2	0.19	0.34
No	18.0		39.2		
Chew/spit tobacco (traditional plus regular) during pregnancy					
Yes	Numbers too small		55.2	0.046	
No			39.7		
Multivitamin or Prenatal vitamin use—one month before pregnancy					
None	22.6	0.32	44.7	0.46	0.91
1–3/week	18.0		42.0		
4–6/week	27.3		25.1		
Daily	15.3		35.9		
Macrosomic ≥4500grams					
Yes	17.1	0.84	51.7	0.46	ND
No	20.0		41.7		
Small for Gestational Age (SGA) (<10 ^th^ %ile)					
Yes	9.7	0.056	44.7	0.87	ND
No	21.4		42.6		
Child ever Breastfed					
Yes	19.4	0.16	42.4	0.88	0.27
No	33.9		40.7		
Child Breastfed (duration in months)					
<6 mo.	22.6	0.01	47.4	0.09	0.10
≥6 mo.	15.9		36.7		

#### Infant Delivery and Feeding Variables

Children who were small for gestational age (SGA) at delivery were less likely than non-SGA children to be obese at age 3 years (9.7% versus 21.4%; p = 0.056) and children who were breastfed for ≥ 6 months were less likely to be obese than children who were not breastfed or who were breastfed for <6 months (15.9% versus 26.6%; p = 0.01) (Table 3). Obese children were breastfed for a shorter number of months compared to non-obese children (mean of 6.60±0.74 months vs. 10.52±0.48 months, respectively; p<0.01).

#### Maternal and Child Psychosocial Variables

Among psychosocial variables, the strongest associations with obesity were found for children born to mothers who were not trying to get pregnant compared to those who were trying (38.5% versus 17.9%; p< 0.01) (Table 4) and the mother experiencing a greater mean number of life stressors during the 12 months before the child was born, (range 0–11) (mean 1.98±0.22 stressors among obese children versus 1.55±0.10 among non-obese children; p = 0.07) (results not shown), although mean number of stressors trended towards statistical significance.

**Table 4:** Percent Obese at Age 3 (≥95^th^ percentile) by Psychosocial Variables, Dietary Intake and Lifestyle Variables.

Lifestyle Variables					
	Non-Native		Alaska Native Population		p-value
	Population		Obese ≥ 95 ^th^ %ile		comparing
	Obese ≥ 95 ^th^ %ile	p-value		p-value	groups
	(N = 96)		(N = 109)		
**Psychosocial variables**					
Maternal postpartum depression diagnosis					
Yes	11.6		36.7		
No	20.2	0.34	43.2	0.66	0.70
Mom diagnosed depression since 3-year-old was born					
Yes	22.4	0.25	31.9	0.56	0.92
No	17.5		43.1		
Child witnessed violence or physical abuse in person					
Yes	29.9	0.62	59.3	0.03	0.85
No	19.8		39.6		
Mother was trying to get pregnant					
Yes	17.9	<0.01	43.7	0.70	0.27
No	38.5		41.3		
**Child beverage consumption at age 3**					
Soda consumption ≥1 cup/day					
Yes	32.0	0.30	41.0	0.24	0.78
No	20.3		57.6		
Soda consumption, cups/day					
0	19.8	0.68	39.2	0.36	0.80
<1 cup	23.9		50.9		
1 cup	35.2		37.1		
≥2 cups	27.4		57.5		
100% fruit juice consumption >1 cup/day					
Yes	25.7	0.06	34.8	0.053	0.01
No	16.8		47.3		
Sweetened or fruit drink ≥1 cup/day					
Yes	21.0	0.91	46.8	0.24	0.50
No	20.5		39.2		
Usually drinks whole milk					
Yes	20.2	0.80	39.3	0.99	0.87
No	19.1		39.4		
**Child Food Consumption at Age 3**					
>2x Candy, cookies or other sweets /day					
Yes	15.2	0.26	48.2	0.38	0.15
No	20.8		41.3		
≥2x vegetables or salad/day					
Yes	17.9	0.24	40.1	0.66	0.60
No	22.9		42.8		
≥2x Fresh, canned, frozen or dried fruit/ day					
Yes	19.6	0.86	38.2	0.18	0.45
No	20.4		46.7		
≥1x French fries/tator tots or potato chips/ day					
Yes	21.8	0.49	49.9	0.02	0.04
No	19.0		35.8		
Child TV viewing					
≤1 hour	20.6	0.80	46.7	0.15	0.43
>1 hour	19.5		37.9		

#### Child Dietary and Lifestyle at 3 years of age

The prevalence of obesity was higher among children who drank more than one cup of 100% fruit juice a day compared to children who drank one cup or less (25.7% versus 16.8%; p = 0.06) although the results were not statistically significant (Table 4). Other dietary variables were less strongly associated with obesity.

#### Multivariate Analysis for Obesity at age 3 years among non-Native Children

After conducting a multivariate analysis process with all variables with a p<0.05 in bivariate analysis and other variables previously associated with increased risk for obesity based on other study findings (e.g. maternal obesity, breastfeeding and 100% juice consumption), increased breastfeeding duration (months) (OR 0.95, 95%CI 0.91–0.91) was protective against childhood obesity at age 3 years in non-Native 3 year old children and low income (<$10,000 in the 12 months before the child was born) was associated with increased risk (OR 2.01, 95%CI 1.01–4.01; Table 5). Maternal pre-pregnancy obesity was also associated with increased risk (OR 2.01, 95%CI 1.01–4.01; Table 5).

**Table 5:** Multivariate Logistic Regression for Obesity at Age 3 among Alaska Non- Native* Children (n = 474).

Variable	Odds Ratio (OR) 95%CI	P value
White race	1.00	
Non-white race	1.46 (0.73–2.92)	0.37
Maternal Pre-pregnancy BMI Category		
Non-obese (<30)	1.00	
Obese (≥30)	1.77 (0.90–3.47)	0.10
Maternal age, years	1.22 (0.93–1.61)	0.15
Trying to get pregnant, pregnancy intention	1.43 (0.71–2.89)	0.31
Breastfeeding, months	0.95 (0.91–0.995)	0.03
Income <$10,000 in 12 months before birth	3.93 (1.22–12.70)	0.02
Mom smoked during post-partum period	1.09 (0.3–2.92)	0.28
Married at birth of child	2.16 (0.93–5.04)	0.07
100% fruit juice consumption >1 cup/day	1.72 (0.91–3.24)	0.10

*Note: As determined by maternal race.

### Childhood Obesity Risk Factors among Alaska Native Children

#### Maternal and Family Socio-Demographic Variables

Among Alaska Native children, obesity risk was greater among residents of the Northern/Southwest region of the state in comparison with other areas, such as Anchorage (51.6% versus 32.4%; p = 0.04). Other variables associated with obesity included rural vs. non-rural birth residence (47.4% versus 32.7%; p = 0.02); paternal education <12 versus >12 years (55.2% versus 21.4%; p = 0.02) (Table 2) and prenatal WIC participation vs. no participation (47.1% versus 31.5%; p = 0.02) A decreased ratio of income/dependents in the 12 months prior to the child’s birth ($8,003.05/dependent±735.41 among obese children versus $8,971.76/dependent±544.94 among non-obese children although results were not statistically significant (p<0.29; results not shown). At 3 years of age, the income by dependent ratio was lower in the families of obese children (for prior 12 months) $7099.82±539,36 versus $8826.84±578.29; p = 0.03; results not shown).

#### Prenatal Maternal Health and Infant Feeding

Prenatal maternal use of chew/spit tobacco vs. no use (55.2% versus 39.7%; p = 0.046) (Table 3) and fewer months of breastfeeding (7.78±0.91 among obese children versus 11.07±0.92 months among non-obese children, p<0.01) (results not shown) were associated with increased risk for obesity at age 3.

#### Child Dietary and Lifestyle at 3 years of age

Among Alaska Native 3 year olds, children who witnessed violence or abuse before age 3 years versus not witnessing either outcome had a greater risk for obesity (59.3% versus 39.6%; p = 0.03) (Table 4) as did those who consumed > 1 cup/day of 100% fruit juice (34.8% compared to 47.3% among children that drank ≤ 1 cup/day (p = 0.053) although results did not meet statistical significance (Table 4)). Children that consumed one or more servings of fried potatoes a day also had an increased obesity prevalence (49.9% versus 35.8%; p = 0.02) (Table 4). When beverage consumption patterns were stratified by region in relation to child obesity status, with beverages defined as milk, soda, water, sweetened drinks and 100% fruit juices, we found much higher consumption patterns of all beverages among residents of the Northern/Southwest region, although only sweetened drinks (p<0.01) and milk (p = 0.03) were significant at the 95% confidence level (Table 6) for Alaska Natives and soda (p = 0.03) and sweetened drinks (p<0.01) for Alaska Natives and Alaska non-Natives (Table 7).

**Table 6:** Percentage of beverage consumption among obese Alaska Native 3 year-olds, by residence region.

	Anchorage	Interior	Northern/Southwest	Gulf Coast/Southeast	P value
**Type of Beverage**					
Whole milk	30.4	27.8	37.8	32.9	0.90
≥1 cup any milk	12.6	61.8	71.3	32.0	0.03
≥1 cup soda	0.0	0.0	13.7	0.0	0.12
>1 cup water	89.5	61.8	76.4	89.1	0.52
≥1 cup sweetened fruit drink	42.3	17.7	73.8	22.8	<0.01
>1 cup 100% fruit juice	45.4	40.3	37.1	35.8	0.92

**Table 7:** Percentage of beverage consumption among all obese Alaskan 3-year-olds (Alaskan Native and Alaskan non-Native), by residence region.

	Anchorage	Interior	Northern/Southwest	Gulf Coast/Southeast	P value
**Type of beverage**					
Any whole milk	33.1	28.5	40.0	36.4	0.83
≥1 cup any milk	26.7	52.4	71.0	53.6	0.06
≥1 cup soda	0.2	7.5	13.2	0.0	0.03
>1 cup water	81.4	51.2	76.5	54.7	0.12
≥1 cup sweetened fruit drink	22.0	19.3	71.4	11.3	<0.01
>1 cup 100% fruit juice	55.8	64.7	37.0	71.0	0.045

#### Multivariate Analysis for Obesity at age 3 years among Native Children

During multivariate analysis, with those variables significant in bivariate at a p<0.05, variables associated with obesity among Alaska Native children were witnessing abuse/domestic violence as a 3 year old (OR 2.98, 95% CI 1.17–7.60; Table 8). Consuming more than 1 cup of 100% fruit juice per day was associated with reduced risk for obesity at age 3 (OR 0.53, 95%CI 0.29–0.99; Table 8).

**Table 8:** Multivariate Logistic Regression for Obesity at Age 3 among Alaska Native* Children (n = 226).

Variable	Odds Ratio (OR) 95%CI	
Maternal age, years	0.83 (0.63–1.11)	0.21
Child witnessed abuse before age 3	2.98 (1.17–7.60)	0.02
Geographical Location		
Anchorage	1.00	
Interior	1.11 (0.43–2.89)	0.83
Northern/Southwest	1.43 (0.63–3.22)	0.39
Gulf Coast/Southeast	0.65 (0.24–1.80)	0.41
Participation in WIC Program Prenatally	1.51 (0.71–3.19)	0.28
Maternal Pre-pregnancy BMI Category		
Non-Obese (<30)	1.00	
Obese (≥30)	1.87 (0.94–3.72)	0.07
Eating fried potatoes at age 3 (yes, any)	1.63 (0.89–2.98)	0.11
Breastfeeding (months)	0.99 (0.96–1.02)	0.44
Maternal chew/spit tobacco use (any) prenatal	1.34 (0.55–3.24)	0.52
Income by dependents at 3 years	0.999986 (0.99993–1.00005)	0.67
100% fruit juice consumption >1 cup/day	0.54 (0.29–0.99)	0.047

*Note: As determined by maternal race.

## Discussion

This cohort of Alaskan children followed from birth through age 3 years provided an opportunity to evaluate early life risk factors for obesity. This is the first statewide study to evaluate factors associated with increased odds of childhood obesity in Alaska and among Alaska Native children, a population group at high risk for obesity [3, 5]. We found a similar percentage of obesity at three years of age (24.9%) to that reported by the Alaska WIC program (which serves low-income children age 2–4 years) (21%) and higher than that reported for kindergarteners during the 2010–11 oral health survey (approximately age 5 years; 16%) [3, 17]. We found a higher percentage of Alaska Native 3 year old children who were obese (42.5%) than the survey conducted by Eberling (24.6%, 95% CI (17.4–33.1), although the latter included a relatively small number of Alaska Native or American Indian subjects (n = 126) so the estimate may not have been as precise and the subjects were older. Other previous reports have not provided estimates of obesity prevalence by race/ethnicity in 3-year old children in Alaska [3]. Previous studies have found that 3 and 4 year old children may have a slighter higher risk for obesity than kindergarten or 5–6 year olds [18] which may explain in part the higher percentage in our 3 year old sample than the study by Eberling et al. Ours is also the first study to document a regional difference in obesity prevalence in 3 year olds with the highest prevalence in the Northern/Southwest part of the state.

### Breastfeeding

Similar to other studies that have focused on early life exposures and risk for obesity in the preschool years [19,20], we found increased breastfeeding duration was associated with lower odds of obesity. In Alaska Native children, however, the relationship between obesity and duration of breastfeeding was no longer statistically significant controlling for other confounders in the multivariate model. It is possible that we lacked statistical power in the multivariate model of Alaska Native children to demonstrate the association of obesity with early termination of breast-feeding found in studies of American Indian children [21]. An alternative hypothesis is that the higher prevalence of obesity in Alaska Native compared to non-Native children results from unmeasured differences in eating patterns and that these may overwhelm any potential benefit from breastfeeding. While we investigated some feeding practices (e.g. fried potato and beverage consumption patterns), future studies should conduct more detailed dietary analysis of early feeding practices in Alaskan families and could consider the contribution of traditional foods as well as non-traditional or modern foods.

### Domestic Violence

We also evaluated the role of exposure to domestic violence and other forms of violence on odds for obesity in early childhood. Our study found an association for a child witnessing abuse or violence in person prior to age 3 years among Alaska Native children in bivariate and multivariate analysis. Other studies have found an association between exposure to chronic intimate partner violence and risk of later childhood obesity at age 5 years [22, 23], including a study, which found that boys exposed to domestic violence before age 5 years, were twice as likely to be obese as adolescents [24]. Exposure to violence may directly impact obesity by causing depression, hopelessness, and subsequently lack of exercise or interest in overall health [25, 26]. Alternatively, this may be a marker for the type of food choices available in that house, the possible presence of fast foods and the overall level of disorder in a child’s life. Future obesity interventions with Alaska Native children, in addition to focusing on diet and exercise alone, may take into consideration the psychosocial context of the family so as to potentially reduce obesity risk.

### Maternal Prenatal Obesity

Among Alaska Native and non-Native children, we found that pre-pregnancy maternal obesity was associated with significantly increased odds for obesity at age 3 years in bivariate analysis although the relationship was attenuated in multivariate and non longer reached statistical significance in Alaska Natives but maintained statistical significance in non-Native children. Previous studies have found that maternal obesity is a significant risk factor for obesity in young children [27, 28]. Our study may have been underpowered to assess any difference based on maternal BMI pre-pregnancy category in Alaska Natives. Addressing maternal obesity prior to pregnancy and even during pregnancy can have inter-generational benefits in terms of reducing overall odds of obesity.

### Sugar Sweetened Beverages, Food Consumption Patterns and Obesity

In bivariate analysis, increased consumption of 100% fruit juice was associated with obesity at age 3 in Alaska non-Natives but protective against obesity in Alaska Natives with similar results in multivariate analysis for Alaskan Native children. It is not clear why 100% fruit juice consumption was protective against obesity in Alaska Native children, although there may have been unmeasured confounding factors or interaction with region of residence that explain this relationship. We did find that increased 100% fruit juice consumption was associated with higher reported intake of fruits and vegetables in Alaska Native children but not non-Natives. It is possible that in this population the consumption of fruit juice may be a marker for reduced caloric intake of energy dense, low nutrient value foods. Increased consumption of fruits and vegetables is associated with reduced risk for obesity in adults, through potentially healthy overall lifestyles [29].

Our findings in Alaska non-Natives are consistent with previous studies that have found increased odds of childhood obesity with higher liquid calorie intake, including 100% fruit juice [30, 11]. On further investigation of the contribution of all energy-dense beverages (including 100% fruit juice), we found a higher frequency of consumption of all beverages in the Northern/Southwest region of the state where obesity prevalence was highest in Alaska Natives, which doesn’t correspond with our finding of a protective association between 100% fruit juice and obesity risk at age 3. Increased energy dense beverage consumption occurred among both obese and non-obese Alaska Native children from this region.

Previous studies suggest that all children [30], including those among indigenous populations [31] should avoid excessive consumption of energy dense, low nutrient value beverages and foods replace these with water and other healthier options. Consumption of water has been found to increase the resting energy expenditure in overweight and obese children [32]. Unfortunately, the Northern/Southwest region has a large number of villages that lack piped water to households [33, 34], and many residents rely on community wells or relatively expensive bottled water.

### Low income

We found a significance increase in risk for obesity among non-Native Alaska children based on low-income status in the 12 months prior to birth. Previous studies in high risk populations have found an association between low household income and increased risk for childhood obesity [35, 36] and studies have warned that interventions that simply counsel individuals on which food types or physical activity regimens are appropriate will not be sustainable in communities that do not have access to these types of foods/beverages or opportunities for physical activity due to poverty [37]. The role of income and family livelihood likely permeates many risk factors for obesity including types of foods and beverages consumed. It is possible we did not find any independent association in Alaska Native children for income status after adjusting for other factors including WIC participation and location of residence, because the distribution of income is narrower in Alaska Native families with most families having a low income at birth (mean for Alaska Natives $22,993.90±814.35 versus $35,657±762.65 for non-Natives) and at 3 years of age (mean for Alaska Natives $30,207.72±1237.16 versus $49,72.34±1145.80 for non-Natives).

### Limitations

Limitations of our study include recall bias (questions were asked retrospectively, both in the postnatal period for PRAMS and for CUBS) as well as self-report and mode bias—some mothers may report certain behaviors more or less willingly than other mothers based on their experiences or they may be more or less willing to disclose certain behaviors during a mail survey [38, 39]. Our outcome of childhood obesity was based on self-reported measurements by the mother and previous studies have found some misclassification of child weight category based on parental report, with under estimates of child heights resulting in upward bias in obesity prevalence estimates [40, 41]. Some factors that we examined had missing values, including 16.5% of respondents missing information on the outcome of childhood BMI and 10.5% missing information on paternal education. When we compared percentages of socio-demographics between those who were missing BMI measurements and those that were not there were no differences in paternal education or child sex, but those who were missing BMI measurements were more likely to have less maternal education, live in rural areas and the Northern/Southwest part of the state and be Alaska Native. There may have been systematic error introduced to our models due to missing information for non-respondents to both surveys (although the analysis weights did attempt to incorporate non-response), and individual question non-response. The number of respondents to both surveys was small, limiting the statistical power of our study to identify significant associations. As both surveys are on-going, further analysis using additional years of data could help to address some of these limitations.

Our sample size to compare differences in risk factors between non-Native and Alaska Native was also small so may not have had sufficient power to detect differences in many cases. Also, as we preformed many statistical tests and many of these statistical associations would be diminished or disappear after multiple comparison adjustment, some associations may have been found by chance and should be replicated in larger studies. As this was a hypothesis generating study with a limited sample size, we chose not to do multiple comparison testing [16], but follow-up studies with larger sample sizes conducted in Alaska that test a high number of predictors should use multiple comparison testing.

## Supporting Information

S1 Appendix Additional Methodological Details on PRAMS and CUBS Variables. (DOC)

S1 Fig Regions of Alaska. (DOCX)
