# Supplementary material for: Risk Factors for Obesity at Age 3 in Alaskan Children, Including the Role of Beverage Consumption: Results from Alaska PRAMS 2005-2006 and Its Three-Year Follow-Up Survey, CUBS, 2008-2009
Source: PLoS One. 2015 Mar 20;10(3):e0118711. doi: 10.1371/journal.pone.0118711 (PMC4368660; doi:10.1371/journal.pone.0118711)
Supplement: S1 Appendix — (DOC) [file pone.0118711.s001.doc]

Appendix S1: Additional Methodological Details on PRAMS and CUBS Variables

*Demographic and Prenatal Risk Factors*

Maternal demographic and pre-pregnancy or prenatal risk factors were from the PRAMS survey or the child’s birth certificate. Factors selected for inclusion in the bivariate analysis included maternal age, education, marital status, socioeconomic status (as measured by an income-to-number of dependents ratio and prenatal participation in the Women, Infants and Children’s Program (WIC)), maternal race, urban/rural residence at time of birth (urban residence included Anchorage, Fairbanks, Juneau, Ketchikan, Matanuska-Susitna, and Sitka Boroughs while the remaining census areas or boroughs were considered rural for this study), pre-existing diabetes mellitus, gestational diabetes mellitus, pre-pregnancy and prenatal smoking, prenatal use of spit tobacco products, weight gain during pregnancy, pre-pregnancy weight category, the number of life stressors experienced by the mother in the year before the child was born (list of 13 events included: close family member was very sick and went to the hospital; separation or divorce from husband/partner; moved to a new address; was homeless; husband/partner lost job; she lost her job; argued with husband/partner more than usual; husband/partner said he didn’t want her to be pregnant; a lot of bills she couldn’t pay; in a physical fight; husband/partner or she went to jail; someone close to her had a bad problem with drinking or drugs; someone close to her died), pre-pregnancy multivitamin or prenatal vitamin use and whether the mother was trying to get pregnant. Income levels (from the 12 months *before* the child was born) were collected as an ordinal variable and used to calculate an income to dependent ratio. The mean value of the income grouping was divided by the number of individuals dependent on that income. Paternal demographic factors included paternal education. For both maternal and paternal education, given the high number of missing values, a missing category was included in the bivariate analysis to evaluate whether there were any significant differences in the prevalence of obesity based on educational level and in comparison with missing.

*Postnatal Maternal and Infant Delivery Risk Factors*

Maternal postnatal risk factors were from PRAMS. Selected factors included cigarette smoking during the postpartum period and postpartum depression diagnosis. Infant delivery variables were taken from the child’s birth certificate and included macrosomic birthweight (≥4500 grams), and small for gestational age (<10%ile) using Center for Disease Control (CDC) reference data sets. Infant postnatal variables included any breastfeeding, as well as duration of breastfeeding, which was collected on CUBS.

*Child risk factors at age 3*

Variables at age 3 from the CUBS survey that were included in the analysis pertained to food and beverage consumption and lifestyle (including frequency of watching TV and exposure to violence or physical abuse). Other factors included region of current residence and whether the mother had been diagnosed with depression since the child was born.
