# Supplementary figures and images for: Risk Factors for Obesity at Age 3 in Alaskan Children, Including the Role of Beverage Consumption: Results from Alaska PRAMS 2005-2006 and Its Three-Year Follow-Up Survey, CUBS, 2008-2009
Source: PLoS One. 2015 Mar 20;10(3):e0118711. doi: 10.1371/journal.pone.0118711 (PMC4368660; doi:10.1371/journal.pone.0118711)

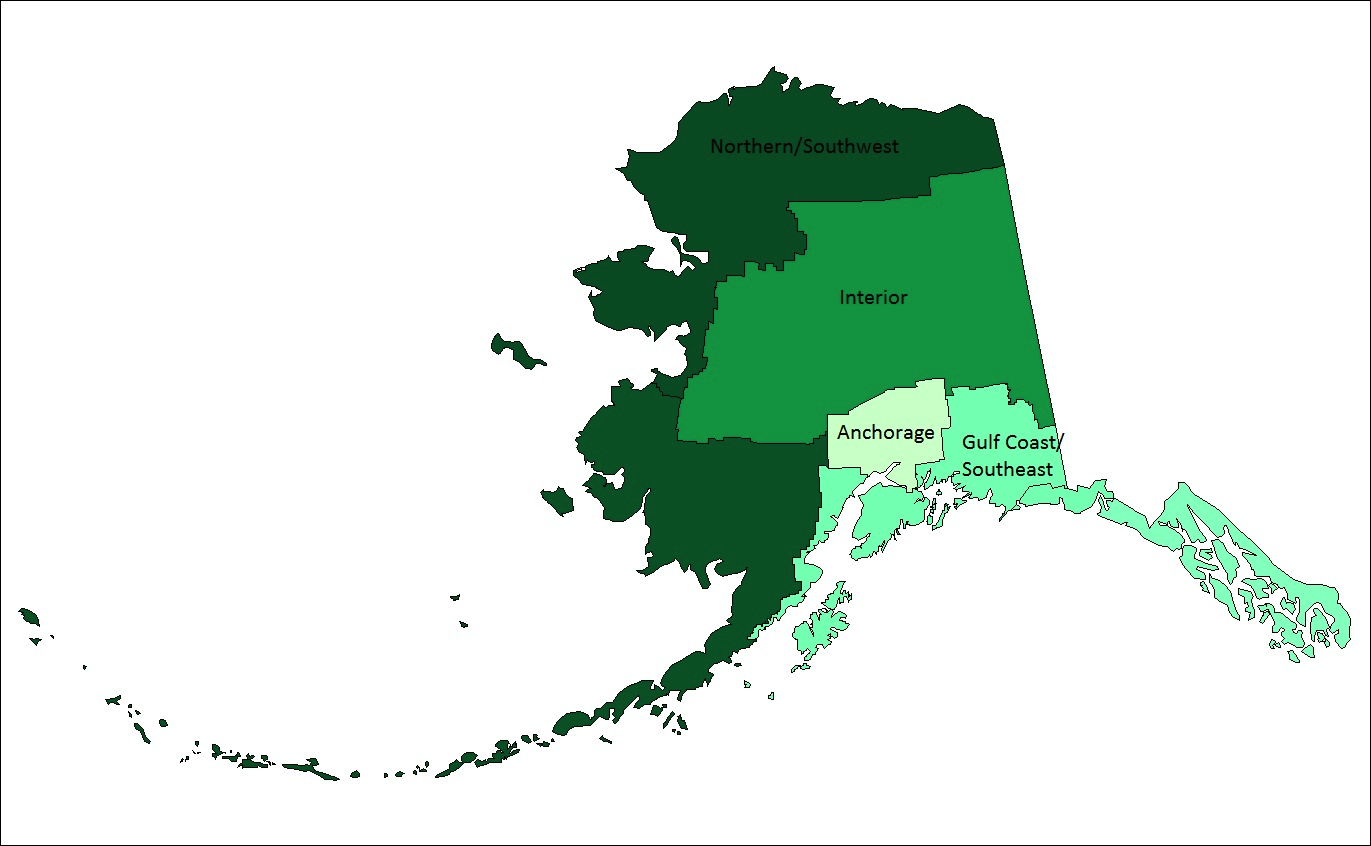


Figure S1: Regions of Alaska

Supplement: S1 Fig — (DOCX) [file pone.0118711.s002.docx]
